# Croonian Lecture

**Published:** 1810-12

**Authors:** William Hyde Woollaston

**Affiliations:** M. D. Sec. R. S.


### Croonian Lecture. By William Hyde Wollaston, M. D. Sec. R. S.

(Phil. Trans. Part I. for 1810.)

THIS Lecture, read before the Royal Society, according to annual custom, and recently published in the Philosophical Transactions, is divided into three parts.

Part I. treats of the duration of *Voluntary Action*.

Part II. attempts to investigate the origin of *Sea-Sickness*, as arising from a simple mechanical cause deranging the circulation of the blood.

Part III. endeavours to explain the advantage derived from riding, and other modes of gestation, in assisting the health under various circumstances, in preference to every species of actual exertion.

### Part I. *On the Duration of Muscular Action.*

THE necessity of occasional intermissions from a series of laborious exersions*, is within the experience of every one; the fatigue of continuing the effort of any one voluntary muscle without intermission even for a few minutes is also sufficiently known; but there is a third view of the duration of muscular action which appears to have escaped the notice of physiologists; for I believe it has not hitherto been observed that each effort, apparently single, consists in reality of a great number of contractions repeated at extremely short intervals: so short indeed that the intermediate relaxation cannot be visible, unless prolonged beyond the usual limits by a state of partial or general debility.

I have been led to infer the existence of these alternate motions from a sensation perceptible upon inserting the extremity of the finger into the ear. A sound is then perceived which resembles most nearly that of carriages at a great distance passing rapidly over a pavement.

* The fact of the muscular fibre becoming debilitated by long action, and then able to resist external impressions only in a lessened degree, we had occasion formerly to notice (*Med. and Phy. Jour. Vol.* 24. *p.* 7) as a circumstance involving much practical utility. Dr. Wollaston's Lecture, though not directly to this point, affords us the opportunity again to turn the attention of the actual practitioner to this principal, or condition of the muscular fibre. In the reduction of dislocations, in the passing sphincters, in overcoming the resistance of muscles on many other occasions, a knowledge of this fact may often afford the means of relief, and sometimes save the reputation of the practitioner. A striking instance of this is noticed in the place referred to, in the first part of this note.

The rapidity of the motion varies according to the degree of force with which the finger is retained in its place. The sound thus perceived is not at all dependent on the degree of pressure upon the tympanum; for, on the contrary, the vibratory sound is most distinct when that pressure is slight, if the finger be at the same time rendered rigid by the forcible action of antagonist muscles; and when the ear is stopped with great force without the presence of muscular action, no such sound is produced. For instance, if the head be rested upon the hand in such a position, as to press with its whole weight upon the ball of the thumb applied to the ear, no noise is perceived, unless the extremity of the thumb be at the same time pressed against the head, or unless the action of some other muscles be communicated to the ear, by any inadvertence in the method of conducting the experiment.

When I endeavoured to estimate the frequency of these vibratory alternations, they appeared to be in general between 20 and 30 in a second; but it is possible that the method I employed may be found defective, and it is to be hoped that my estimate may be corrected, by some means better adapted to the determination of intervals that cannot actually be measured.

It was by imitation alone that I was enabled to judge of their frequency. For this purpose I contrived to render the vibration itself, and the imitative sound, both audible by the same ear.

While my ear rested on the ball of my thumb, my elbow was supported by a board lying horizontally, in which were cut a number of notches of equal size, and about $\frac{1}{7}$ of an inch asunder. Then, by rubbing a pencil or other round piece of wood with a regular motion along the notches, I could imitate pretty correctly the tremor produced by the pressure of my thumb against my head, and by marks to indicate the number of notches passed over in 5 or 10 seconds, observed by my watch, I found repeated observations agree with each other as nearly as could be expected; for I could not depend upon exerting the same degree of force in different trials.

That I might not be deceived by the resemblance of tremors, which coincided only at alternate beats, and therefore might be considered as octaves in music to each other, I sometimes employed notches at greater and sometimes at less distances from each other, but the result was nevertheless the same; and in order to avoid any error that might be caused by some accidental quality of the sound arising from the length of the muscle employed, or length of the bones concerned in conveying the imitative sound to my ear, I made the following variation of the experiment. My ear was stopped by a cushion pressed upon by the end of a notched stick that rested on my foot, and thus conveyed the vibration from the muscles of my leg to the ear, along with the tremor produced by friction upon the notches; and still the results were nearly the same; varying in frequency between 20 and 30 in a second, according to the degree of force exerted in the experiment.*

As a further proof that I was not much deceived in my judgment of the frequency of these vibrations, I requested two or three of my friends to repeat the same experiment for me, and our agreement was such as to confirm me in the opinion, that there could be no very considerable error in the estimate.

The greatest frequency that I think I have observed, was about 35 or 36 in a second, and the least was as low as 14 or 15; but in attempting to lessen the number of vibrations, there appears to be a degree of unsteadiness which prevents any accurate measurement of the real number.

It is very probable, that in cases of great debility the number may be even considerably less, and may be the reason of that visible unsteadiness, which is known to occur in persons enfeebled by age, or much reduced by disease.

Possibly the foregoing observation may not be altogether new to some members of this Society, as it is now about 17 or 18 years since it first occurred to me, and I was then accustomed occasionally to mention it in conversation with my friends; but I am not aware that any other person has made the same remark respecting the vibratory nature of muscular action, although I find that Grimaldi had observed the sound that occurs upon stopping the ears, but ascribed it, according to the notions that prevailed in his time, to the hurried motions of the animal spirits.†

---

* The resemblance of the muscular vibrations to the sound of carriages at a distance, I apprehend to arise not so much from the quality of the sound as from an agreement in frequency with an average of the tremors usually produced by the number of stones in the regular pavement of London, passed over by carriages moving quickly.

If the number of vibrations be supposed 24 in a second, and the breadth of each stone be about 6 inches, the rate of a carriage thus estimated would be about 8 miles an hour, which agrees with the truth as nearly as the assumptions on which the estimate is founded.

† Vera itaque ratio experimenti prædicti est, quia in digito et brachio totoque corpore continuato fiunt multi motus ac tremores, ob spirituum agitationem huc illuc perpetuo accurrentium.

Grimaldi, Physicomathesis de Lumine, p. 383.

PART II. *On Sea-Sickness.*

THE cause of sea-sickness has not hitherto been fully explained; and although the explanation which I am about to propose, may not appear altogether satisfactory to persons who, when at sea, are also rendered giddy by the incessant motion of the waves, and are consequently liable to consider as cause and effect phænomena which in their minds are constantly associated, yet the observation on which it is founded may deserve to be recorded, on account of the degree of relief that may be obtained in that most distressing affection.

After I had been harassed by sea-sickness during a short voyage for some days, and had in vain attempted to account for the difference between the inexperienced passenger, and those around him more accustomed to the motion of the sea, I imperceptibly acquired some power of resisting its effects, and had the good fortune to observe a peculiarity in my mode of respiration, evidently connected with the motion of the vessel, but of which, in my then enfeebled state, I was unable to investigate either the cause or consequence. In waking from a state of very disturbed sleep, I found that my respirations were not taken with the accustomed uniformity, but were interrupted by irregular pauses, with an appearance of watching for some favourable opportunity for making the succeeding effort; and it seemed as if the act of inspiration were in some manner to be guided by the tendency of the vessel to pitch with an uneasy motion.

The mode by which I afterwards conceived that this action could primarily affect the system, was by its influence on the motion of the blood; for, at the same instant that the chest is dilated for the reception of air, its vessels become also more open to the reception of the blood, so that the return of blood from the head is more free than at any other period of a complete respiration. On the contrary, by the act of expelling air from the lungs, the ingress of blood is so far obstructed, that, when the surface of the brain is exposed by the trepan, a successive turgescence and subsidence of the brain is seen, in alternate motion with the different states of the chest. It is probably from this cause that, in severe head-aches, a degree of temporary relief is obtained by occasional complete inspirations.

In sea-sickness also the act of inspiration will have some tendency to relieve, if regulated so as to counteract any temporary pressure of blood upon the brain; but the cause of such pressure requires first to be investigated.

All those who have ever suffered from sea-sickness (without out being giddy) will agree that the principal uneasiness is felt during the subsidence of the vessel by the sinking of the wave on which it rests. It is during this subsidence that the blood has a tendency to press with unusual force upon the brain.

If a person be supposed standing erect upon deck, it is evident that the brain, which is uppermost, then sustains no pressure from the mere weight of the blood, and that the vessels of the feet and lower parts of the body must contract, with a force sufficient to resist the pressure of a column of blood, of between five and six feet from the head downwards.

If the deck were, by any means, suddenly and entirely removed, the blood would be no longer supported by its vessels; but both would fall together with the same velocity by the free action of gravity; and the same contraction of the vessels which before supported the weight of the blood would now occasion it to press upon the brain, with a force proportional to its former altitude.

In the same manner, and for the same reason, during a more gradual subsidence of the deck, and partial removal of support, there must be a partial diminution of the pressure of the blood upon its vessels, and consequently, a partial re-action upon the brain, which would be directly counteracted by a full inspiration.

The consequence of external motion upon the blood will be best elucidated by what may be seen to occur in a column of mercury similarly circumstanced.

A barometer, when carried out to sea in a calm, rests at the same height at which it would stand on shore; but, when the ship falls by subsidence of the wave, the mercury is seen apparently to rise in the tube that contains it, because a portion of its gravity is then employed in occasioning its descent along with the vessel; and accordingly, if it were confined in a tube closed at bottom, it would no longer press with its whole weight upon the lower end. In the same manner, and for the same reason, the blood no longer presses downwards with its whole weight, and will be driven upwards, by the elasticity which before was merely sufficient to support it.

The sickness occasioned by swinging is evidently from the same causes as sea-sickness, and that direction of the motion which occasions the most piercing sensation of uneasiness, is conformable to the explanation above given.

It is in descending forwards that this sensation is perceived; for then the blood has the greatest tendency to move from the feet towards the head, since the line joining them is in the direction of the motion. But when, in the descent backwards, the motion is transverse to the line of the body, it it occasions little comparative inconvenience, because the tendency to propel the blood towards the head is then inconsiderable.

The regularity of the motion in swinging, afforded me an apparently favourable opportunity for trying the effect of inspiration; but although the advantage was manifest, I must confess, it did not fully equal the expectations I had formed from my experience at sea. It is possible, that the suddenness of the descent may in this case be too great to be fully counteracted by such means; but I am inclined to think that the contents of the intestines are also affected by the same cause as the blood: and if these have any direct disposition to regurgitate, this consequence will be in no degree counteracted by the process of respiration.

A friend of mine informed me that he had endeavoured to counteract this mechanical effect upon the stomach, and had experienced immediate relief from a slight degree of sea-sickness, by lying down upon the deck with his head towards the stem of the vessel; by means of which, upon pitching he was in the attitude of a person descending backwards in a swing.

Whether the stomach be or be not thus primarily affected, or only by sympathy with the brain, the sensation of sinking is in all cases referred directly to the stomach, which is seized with such instantaneous retching, that no person, who has not been so situated, can form a just conception of it.*

In thus referring the sensations of sea-sickness in so great a degree, to the agency of mere mechanical pressure, I feel confirmed by considering the consequence of an opposite motion, which by too quickly withdrawing blood from the head, occasions a tendency to faint, or that approach to fainting, which amounts to a momentary giddiness with diminution of

* There is one occasion upon which a slighter sensation of this kind is perceived, and it appears to indicate the direction of the motion from which it arises, to be downwards. "In a country subject to frequent returns of earthquakes" it is said* that "a few minutes before any shock came, many people could foretel it by an alteration in their stomachs; an effect which" (it is added) "always accompanies the wave-like motion of earthquakes, when it is so weak as to be uncertainly distinguishable."

(Mitchell, Phil. Trans. Vol. LI. 610.)

It seems that the vapours to which these tremendous concussions are owing, immense in quantity, and of prodigious force, being for a time confined on all sides, elevate the surface of a country to a vast extent until they either find vent, or meet with some partial cause of condensation, and hence the alternate heaving and subsidence of the ground, will produce much the same effects as the rising and falling of the swell at sea.

* Phil. Trans. Vol. XLII. p. 41.

mucular muscular power. At a time when I was much fatigued by exercise, I had occasion to run to some distance, and seat myself under a low wall for shelter from a very heavy shower. In rising suddenly from this position I was attacked with such a degree of giddiness, that I involuntarily dropped into my former posture, and was instantaneously relieved, by return of blood to the head, from every sensation of uneasiness.

Since that time, the same affection has frequently occurred to me in slighter degrees, and I have observed, that it has always been under similar circumstances of rising suddenly from an inclined position, after some degree of previous fatigue. Sinking down again immediately removes the giddiness; and then, by rising a second time more gradually, the same sensation is avoided.

### PART III. *On the salutary Effects of Riding, and other Modes of Gestation.**

IN the preceding instances of disturbing the circulation of the blood, by external motion, the effect is disagreeable, and proportionally prejudicial. There may indeed be cases of disorder, in which it will be salutary, but these are probably less frequent than is generally supposed.

In the observations which follow, general opinion will concur with me, on the benefit derived from external, or passive motion, and I hope, that in ascribing its good effects to their true cause, I shall enable others to make a valuable distinction, which has not yet been preserved with due care, between one motion which is salutary, and another which is very frequently pernicious. For, although the term *gestation* is employed by medical writers, as a general term comprehending riding on horseback, or in a carriage, and although the merits of such motions, especially the former, were clearly noticed, and perhaps even over-rated, by the discernment of SYDENHAM, I believe that no explanation has yet been given, of the peculiar advantages of external motion, and am persuaded, that the benefits to be derived from carriage exercise are by no means in so high estimation as they ought to be.

---

* Our readers will, perhaps, recollect an Inaugural Dissertation, *de Typhi Remediis*, in 1807 by Dr. Baldwin Wake, in which the beneficial effects of *gestation*, in fever, are particularly detailed. Many strong facts are there given in support of *gestation* as a remedial process in certain states of fever. We then thought, as we now do, not only from the relation of Dr. Wake, but from some facts that have come particularly under our notice, that gestation may be used with much advantage, in some morbid states: but that further observations are required to fully ascertain the causes and periods of disease suited to it, with a precision that may enable practitioners to employ it without hazard, and with a reasonable prospect of success.

Under

Under the common term *exercise*, active exertion has too frequently been confounded with passive gestation, and fatiguing efforts have consequently been substituted for motions that are agreeable, and even directly invigorating, when duly adapted to the strength of the invalid, and the peculiar nature of his indisposition.

The explanation which I am about to offer of the effects of external motion upon the circulation of the blood, is founded upon a part of the structure observable in the venous system, the mechanical tendency of which cannot be doubted. The valves which are every where dispersed through those vessels, allow free passage to the blood, when propelled forward, by any motion that assists its progress ; but they oppose an immediate obstacle to such as have a contrary tendency. The circulation is consequently helped forward by every degree of gentle agitation. The heart is supported in any laborious effort that may have become necessary, by some obstacle to its exertions ; it is assisted in the great work of restoring a system, which has recently struggled with some violent atrack ; or it is allowed, as it were, to rest from a labour, to which it is unequal, when the powers of life are nearly exhausted by any lingering disorder.

In the relief thus afforded to an organ so essential to life, all other vital functions must necessarily participate ; and the various offices of secretion, and assimilation, by whatever means they are performed, will not fail to be promoted during such comparative repose from laborious exertion.

Even the powers of the mind itself, though apparently, least likely to be influenced by mere mechanical means, are manifestly, and in many persons, most immediately affected, by these kinds of motion.

It is not only in cases of absolute deficiency of power to carry on the customary circulation, that the beneficial effects of gestation are felt, but equally so, when comparative inability arises from redundancy of matter to be propelled. When from fulness of blood, the circulation is obstructed, the whole system labours under a feeling of hurry and agitation, with that sensibility to sudden impressions, which is usually termed nervousness. The mind becomes incapable of any deliberate consideration, and is impressed with horrors that have no foundation, but in a distempered imagination.

It is in moderate degrees of this species of affection, that the advantages of carriage exercise are most sensibly felt. The composed serenity of mind that succeeds to the previous alarm, is described by some persons with a degree of satisfaction, that evinces the decided influence of the remedy. With this steadier tone of mind, returns its full power of cool reflection; reflection; and if the imagination becomes more alive than usual, its activity is now employed in conceiving scenes that are amusing, and agreeable.

As an instance of direct relief to a circulation, labouring from mere fulness of blood, I may adduce that of a person, whose friends, as well as himself, were apprehensive, from the violent and visible throbbing of his heart, of the existence of some organic mischief, and were in some measure alarmed for the consequences.

He was persuaded, and not reluctantly, to go without delay, for medical advice, and was accordingly conveyed in a carriage to the house of some physician of eminence, but did not succeed in finding him at home. As the symptoms did not appear to admit of delay, and were at least not aggravated by the motion, it was hoped that the wished-for advice might be obtained at a part of the town, which happened to be at some distance. But the second attempt proved as fruitless as the former, and a third was made with the same event. Since the throbbing had by that time considerably abated, he was contented to postpone any further efforts to the following day, and directed the carriage homewards. By the time that he returned to his friends, he found, that the motion of travelling over several miles of pavement, had apparently removed the complaint. The pulsation of the heart and arteries had subsided to their natural standard, and he congratulated himself, that his search of a remedy had not been ineffectual, although he had been disappointed as to the source from which he thought he had most reason to expect relief.

If vigour can in any instance be directly given, a man may certainly be said to receive it in the most direct mode, when the important service of impelling forward the circulation of his blood is performed for him by external means. The main spring, or first mover of the system, is thereby, as it were, wound up; and although the several subordinate operations, of so complicated a machine, cannot be regulated in detail, by mere external agency, they must each be performed with greater freedom, in consequence of this general supply of power.

In almost every treatise on the subject of chronical diseases, are to be found numerous instances of the benefit, produced by the several modes of gestation, which have been most generally adopted; as riding on horseback, in carriages, sea-voyages, and swinging. And in many cases, which might be adduced, it has appeared too clear, to admit of a doubt, that the cure of the patient has been owing *solely* to the external agitation of his body, which must be allowed, at least, to have had the effect above explained: that of relieving the

 heart heart and arteries from a great part of their exertion in propelling the blood, and *may* therefore have contributed to the cure, by that means only.

The different modes above mentioned, are adapted from their nature to different degrees of bodily strength, and if there are cases in which, that which appears most eligible, may not suit the situation, or circumstances of the patient, it can not be difficult to contrive other means of giving motion, so as least to incommode, and yet to give the greatest relief. A very gentle and long continued, or even incessant motion, may suit some cases better than any more violent and occasional agitation; and in this way, probably, it is, that sea voyagss have sometimes been attended with remarkable advantage.

